# Efficient Phage Display with Multiple Distinct Non‐Canonical Amino Acids Using Orthogonal Ribosome‐Mediated Genetic Code Expansion

**DOI:** 10.1002/anie.201902658

**Published:** 2019-07-04

**Authors:** Benjamí Oller‐Salvia, Jason W. Chin

**Affiliations:** ^1^ Medical Research Council Laboratory of Molecular Biology Francis Crick Avenue Cambridge CB2 0QH UK

**Keywords:** biorthogonal reactions, cyclopropene, phage display, protein engineering, site-specific bioconjugation

## Abstract

Phage display is a powerful approach for evolving proteins and peptides with new functions, but the properties of the molecules that can be evolved are limited by the chemical diversity encoded. Herein, we report a system for incorporating non‐canonical amino acids (ncAAs) into proteins displayed on phage using the pyrrolysyl‐tRNA synthetase/tRNA pair. We improve the efficiency of ncAA incorporation using an evolved orthogonal ribosome (riboQ1), and encode a cyclopropene‐containing ncAA (CypK) at diverse sites on a displayed single‐chain antibody variable fragment (ScFv), in response to amber and quadruplet codons. CypK and an alkyne‐containing ncAA are incorporated at distinct sites, enabling the double labeling of ScFv with distinct probes, through mutually orthogonal reactions, in a one‐pot procedure. These advances expand the number of functionalities that can be encoded on phage‐displayed proteins and provide a foundation to further expand the scope of phage display applications.

Phage display is a powerful approach for selecting peptides and proteins, from diverse libraries, with high affinity for a molecular target^1^ and has been extensively used to select therapeutic peptides and proteins.^2^ Several adaptations of phage display have expanded its scope for diverse applications, including: the selection of bicyclic peptides constrained by covalent tethering to a small molecule,^3^ the evolution of catalytic function,^4^ the synthesis of new materials and nanowires,^5^ and the profiling of cell‐surface proteomes.^6^

Classical phage display is limited to encoding proteins containing the 20 canonical amino acids, and this limits the range of functions that can be accessed in phage‐displayed peptides and proteins. Selenocysteine can be incorporated but this approach is limited in chemical scope and the sequences that may be encoded.^7^ Close analogues of methionine can be incorporated by selective pressure incorporation; however, this leads to insertion of the analogues in response to all Met codons.^8^

Advances in genetic‐code expansion^9^ have enabled the site‐specific co‐translational incorporation of non‐canonical amino acids (ncAAs) into proteins displayed on phage.4b, 10 These approaches have primarily used variants of the *Menthanococus janaschii* (*Mj*) tyrosyl‐tRNA synthetase (*Mj*TyrRS)/tRNA_CUA_ pair, which is orthogonal in *Escherichia coli*.^10^ This system has been used to label proteins expressed on phage through azide‐alkyne cycloadditions4b, 10a and Staudinger ligations;10b it has also been used to evolve single‐chain antibody variable fragments (ScFvs) with chemical warheads10c, 10d and proteins that chelate metal ions.10f Despite these important advances, current approaches have significant limitations. First, the *Mj*TyrRS/tRNA_CUA_ pair has been exclusively used to incorporate amino acids derived from phenylalanine; this precludes the genetic encoding of diverse aliphatic ncAAs. Second, each phage generated using this approach only incorporates a single type of ncAA in response to a single amber codon in the gene of interest; this precludes the incorporation of multiple distinct ncAAs on a single phage, which may facilitate a range of applications, including the selective double labeling of displayed proteins.

Herein, we report a phage display system for incorporating ncAAs into proteins displayed on phage that takes advantage of the pyrrolysyl‐tRNA synthetase (PylRS)/tRNA pair (Figure 1 a). This pair has been extensively developed for incorporating diverse aliphatic ncAAs into proteins,^11^ and read‐through of stop codons in gene 3 of M13 phage using this pair has been demonstrated as part of a continuous evolution strategy.^12^ However, a phage display system that takes advantage of PylRS/tRNA has not been characterized. Here we demonstrate the site‐specific incorporation of a cyclopropene‐containing ncAA (CypK, N^*ϵ*^‐[((2‐methylcycloprop‐2‐en‐1‐yl)methoxy)carbonyl]‐l‐lysine) into proteins displayed on phage using the PylRS/tRNA pair. Phage‐displayed proteins incorporating CypK are labeled through a rapid inverse electron‐demand Diels–Alder reaction with tetrazine derivatives.^13^ We show that the efficiency of displaying proteins containing ncAAs is substantially improved by translation of the displayed protein fusion from an orthogonal ribosome binding site using an evolved orthogonal ribosome, and that the optimized system can be used to incorporate CypK at diverse sites on an ScFv, in response to both amber and quadruplet codons. Finally, we demonstrate that PylRS/tRNA_UACU_ and an evolved *Mj*TyrRS/tRNA_CUA_ pair can be used to incorporate both CypK and *p*‐propargyloxy‐l‐phenylalanine (PrpF) on an ScFv. This enables the double labeling of the displayed ScFv with distinct probes, through mutually orthogonal reactions, in a one‐pot procedure.

**Figure 1:**
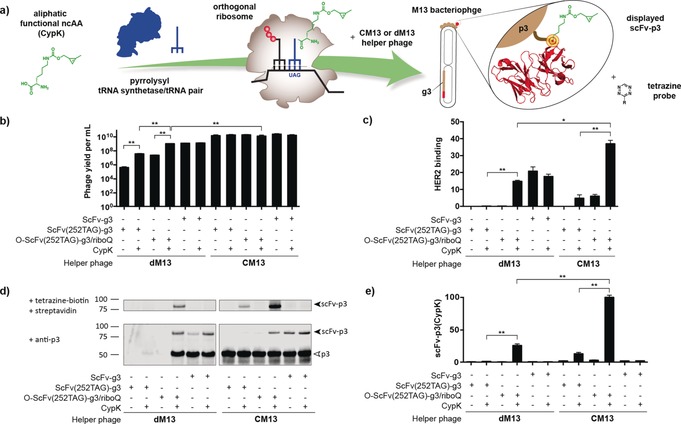
Orthogonal ribosome RiboQ1 enhances incorporation of ncAA in M13 bacteriophage using CM13 and dM13 helper phage. a) ncAA incorporation in a phage‐displayed scFv b) Phage titers in cfu mL^−1^ c) HER2 ELISA signals are shown as a percentage of that for phage expressing WT scFv produced with CM13. d) Western blot fluorescence shows ScFv–p3 labeled with tetrazine–biotin and probed with streptavidin, and ScFv–p3 and p3 probed with an anti‐p3 antibody. e) Quantification of ScFv–p3 labeled with tetrazine–biotin and probed with streptavidin. Data are shown as a percentage of ScFv–p3 detected using RiboQ1 and CM13 in the presence of CypK. Experiments in panels c–e were performed with equal volumes of phage, and inputs were not normalized for titer. All error bars represent the standard error from three biological replicates. (*p<0.01, **p<0.001).

We first developed a phage display system for incorporating ncAAs into phage‐displayed proteins using the PylRS/tRNA pair. This phagemid‐based system is composed of two plasmids (System 1 in Figure S1 in the Supporting Information). The PylRS/tRNA_CUA_ pair is expressed from a high copy pAux plasmid^14^ (Supporting Information, Figure S2), while a phagemid vector is used to encode the gene of interest (herein an anti‐Her2 ScFv) containing an amber codon fused to the p3 gene (g3). We initially introduced an amber codon at position 252 in the scFv (118 EU numbering), creating an ScFv(252TAG)–g3 gene; this site is commonly used to modify antibodies without affecting antigen binding.^15^

Production of phage from cells bearing phagemid‐based systems is dependent on helper phage infection to provide phage coat proteins.1b, 2b All^10^ but one4b report of using *Mj*TyrRS/tRNA_CUA_ derivatives to incorporate ncAAs rely on a helper phage in which g3 has been deleted (dM13); this makes the production of phage particles dependent on expression of the full length p3 fusion from the phagemid vector and phage titers are ncAA dependent in these systems.

We observed CypK‐dependent production of phage upon addition of dM13 to cells bearing our two‐plasmid system with phagemid‐encoded ScFv(252TAG)–g3; phage production increased 100‐fold upon addition of CypK to cells (Figure 1 b). However, the absolute titer of phage from cells bearing ScFv(252TAG)–g3 and receiving CypK, was 30‐fold lower than the titer from cells in which the scFv–g3 fusion did not contain an amber stop codon (3.9×10^7^ vs. 1.2×10^9^ cfu mL^−1^; Figure 1 b). Indeed, we could not easily detect the displayed scFv by enzyme‐linked immunosorbent assay (ELISA; Figure 1 c) nor could we easily detect CypK labeling (Figure 1 d).

We hypothesized that the decreased titer of the phage incorporating CypK in ScFv(252TAG)–g3, with respect to phage produced from scFv–g3 without an amber stop codon, was a result of sub‐optimal read‐through of the amber stop codon. We have previously shown that the efficiency of ncAA incorporation can be enhanced by translating the message of interest, under the control of an orthogonal ribosome binding site, using an evolved orthogonal ribosome (riboQ1).^16^

We therefore created a second‐generation system (System 2 in Figure S1 in the Supporting Information) in which riboQ1 was used to translate O–ScFv(252TAG)–g3 (a phagemid in which ScFv(252TAG)–g3 is downstream of an orthogonal ribosome binding site). Using this system with dM13 helper phage led to CypK‐dependent phage production, with titers an order magnitude higher than in our initial system (Figure 1 b). Moreover, when using System 2 with dM13 for CypK incorporation, the phage titer from the O–ScFv(252TAG)–g3 fusion was comparable to that for the amber‐codon‐free gene fusion translated from a canonical ribosome binding site (Figure 1 b). We suggest that this system maximizes titers by providing sufficient ScFv–p3 containing the ncAA.

The new system allowed us to easily detect the displayed anti‐Her2 scFv, providing an ELISA signal at least 10‐fold above the initial system, and comparable to that from control experiments with amber‐codon‐free ScFv–g3 fusions (Figure 1 c). System 2 also allowed us to easily detect labeling of CypK incorporated into the ScFv–p3 fusion (expressed from O–ScFv(252TAG)–g3), providing a labeling signal at least 10‐fold higher than the initial system (Figure 1 d,e). As expected, both the ELISA and labeling signal were strongly CypK dependent. Moreover, at least 84±6 % of phage displayed reactive CypK as quantified through a biotin‐capture assay^17^ (Supporting Information, Figure S3). We conclude that System2/dM13 enables the specific incorporation of CypK with titers of phage displaying ncAAs that are comparable to the parental system with no amber codon.

Next, we tested an interference‐resistance helper phage (CM13) in combination with System 1. CM13 provides a copy of g3, such that the production of infectious phage particles is not dependent on the phagemid‐encoded p3 fusion. The use of interference‐resistant helper phage commonly leads to the production of high phage titers and facilitates monovalent display.^18^ As expected, we found that phage titers with CM13 helper phage were excellent (10^10^ cuf mL^−1^), and independent of the amber stop codon in ScFv, or the addition of CypK (Figure 1 b). Using the amber codon variant of System 1 (Supporting Information, Figure S1) with CM13 we detected the displayed ScFv and the labeling of CypK (Figure 1 c–e).

The amber codon variant of System 2 displayed the ScFv at approximately 40 % of the level of its no amber version, when using CM13 (Figure 1 c). Monovalent display was confirmed (Supporting Information, Figure S4). Moreover, this system led to the highest level of CypK labeling signal (Figure 1 d,e and Supporting Information, Figure S5), leading to a 7‐fold increase in labeling with respect to the corresponding System 1 experiment. At least 81±8 % of the phage displaying ScFv bears CypK (Supporting Information, Figure S3). We decided to use System 2 with CM13 helper phage for all further experiments.

Next, we demonstrated that System 2 with CM13 enabled ncAA incorporation at diverse sites on phage‐displayed proteins in response to amber or quadruplet codons. We targeted residues far from the paratope (G128, 252) that may be derivatized without affecting binding, as well as the complementarity‐determining regions (CDRs) and their proximity (K161, N186, D233). We observed CypK‐dependent production of the ScFv–p3 fusion protein for each amber mutant (Figure 2 a), which was supported by ELISAs (Supporting Information, Figure S6). Labeling of the fusions confirmed that all the sites tested are accessible for the reaction (Figure 2 a).

**Figure 2:**
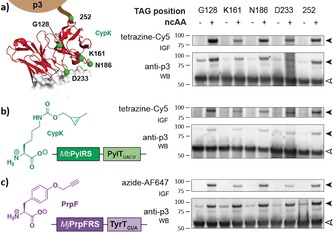
Characterization of flexibility of position, codon, and ncAA. a) CypK is incorporated in response to amber codons at five distinct positions throughout the scFv. The five residues in the ScFv that are targeted for mutagenesis are shown in green (PDB: 1N8Z). CypK was labeled with tetrazine–sulfocyanine‐5 (tetrazine–Cy5). b) Replacing TAG with AGTA in the phagemid and using a Pyl‐tRNA derivative with an extended (UACU) anticodon enables CypK incorporation via quadruplet decoding. c) Adding *Mj*PrpFRS in pAux enables the incorporation of PrpF, which was labeled with azide–AlexaFluor 647 (azide–AF647). Black and white arrows indicate scFv–p3 and p3, respectively. IGF: in‐gel fluorescence. WB: western blot.

We were curious whether we could extend System 2 to enable ncAA incorporation in response to quadruplet codons.^16^ We therefore replaced Pyl‐tRNA_CUA_ in System 2 with an evolved derivative bearing an extended anticodon and introduced the corresponding codons into the ScFv gene. Using this system, we observed CypK‐dependent ScFv–p3 production and labeling at all positions tested (Figure 2 b). These experiments demonstrated that the second‐generation phage display system we have created enables ncAA incorporation using the PylRS/tRNA pair at diverse sites in response to quadruplet codons.

Next, we demonstrated that we could adapt System 2 for incorporation of ncAAs using a different orthogonal aaRS/tRNA pair. To achieve this, we replaced PylRS/tRNA_CUA_ with *Mj*PrpRS/tRNA_CUA_ that directs the incorporation of PrpF (Supporting Information, Figure S1).^19^ ScFv–p3 synthesis was dependent on PrpF, and we labeled the incorporated PrpF with an azide fluorophore, via a Cu^I^‐catalyzed 3+2 cycloaddition (Figure 2 c).

Finally, we asked whether we could combine our advances to facilitate the display and labeling of proteins containing multiple distinct ncAAs. We have previously demonstrated that PylRS/tRNA pairs and *Mj*TyrRS/tRNA‐derived pairs are mutually orthogonal16a and that we can selectively label encoded CypK and PrpF in proteins.^20^ We created a version of System 2 that expresses PylRS/tRNA_UACU_ and *Mj*PrpRS/tRNA_CUA_, and an O–ScFv(127TAG, 252AGTA)–g3 cassette (Supporting Information, Figure S1). Phage produced from this system in the presence of both CypK and PrpF were selectively labeled by both tetrazine–fluorophore conjugates and azide–fluorophore conjugates (Figure 3). The majority of the phage displaying ScFv were labeled with both fluorophores and contain both ncAAs (Supporting Information, Figure S7), and control experiments confirmed the specificity of these labeling reactions (Supporting Information, Figure S8).

**Figure 3:**
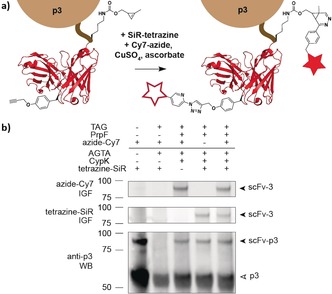
Concerted dual modification of scFv–p3. a) Schematic representation of the one‐pot labeling of an scFv (PDB: 1N8Z) bearing CypK and PrpF. b) In‐gel fluorescence of scFv–p3 dually modified with tetrazine–silicon–rhodamine (tetrazine–SiR, middle, Cy5 channel) and picolylazide–sulfocyanine‐7 (azide–Cy7, top, Cy7 channel) after pulldown via the HA (human influenza hemagglutinin) tag. Western blot (bottom) probed with anti‐p3 shows p3 and scFv–p3. IGF: in‐gel fluorescence. WB: western blot.

In conclusion, we have developed an efficient phage display system for incorporating ncAAs into proteins displayed on phage using PylRS/tRNA. Since this pair can be used to encode multiple, structurally and functionally diverse ncAAs, our system expands the range of ncAAs that may be encoded in proteins displayed on phage. Using this system, we have encoded CypK, which enables the labeling of phage‐displayed proteins, through a bioorthogonal metal‐free cycloaddition, orders of magnitude faster than chemical reactions with functional groups that were previously displayed on phage.4b, 10b The second‐generation system we have developed takes advantage of translation by an evolved orthogonal ribosome to enhance the efficiency with which proteins containing ncAAs can be displayed on phage. The wild‐type, or near wild‐type, display levels we have achieved will facilitate directed evolution experiments (Supporting Information, Figure S4). Modular alterations to our system enable the incorporation of different classes of ncAAs, using distinct orthogonal aaRS/tRNA pairs, and facilitate the incorporation of ncAAs in response to amber or quadruplet codons. Moreover, our approach enables the genetic encoding of multiple distinct ncAAs and enables the one‐pot, site‐specific, dual labeling of phage‐displayed proteins.

Recent exciting advances in the generation and discovery of new mutually orthogonal synthetase/tRNA systems^21^ may enable additional functionalities to be encoded on phage‐displayed proteins. We anticipate that increasing the chemical functionalities, and number of new building blocks, that can be encoded will further expand the scope of phage display to facilitate diverse applications.

## Conflict of interest

The authors declare no conflict of interest.

## Supporting information

As a service to our authors and readers, this journal provides supporting information supplied by the authors. Such materials are peer reviewed and may be re‐organized for online delivery, but are not copy‐edited or typeset. Technical support issues arising from supporting information (other than missing files) should be addressed to the authors.

Supplementary
