# Supplementary material for: Efficient Phage Display with Multiple Distinct Non‐Canonical Amino Acids Using Orthogonal Ribosome‐Mediated Genetic Code Expansion
Source: Angew Chem Int Ed Engl. 2019 Jul 4;58(32):10844–8. doi: 10.1002/anie.201902658 (PMC6771915; doi:10.1002/anie.201902658)
Supplement: Supplementary file 1 — Supplementary [file ANIE-58-10844-s001.pdf]

Angewandte Chemie
Eine Zeitschrift der Gesellschaft Deutscher Chemiker

Supporting Information

Efficient Phage Display with Multiple Distinct Non-Canonical Amino Acids Using Orthogonal Ribosome-Mediated Genetic Code Expansion

*Benjamí Oller-Salvia and Jason W. Chin**

anie_201902658_sm_miscellaneous_information.pdf

Table of Contents

Experimental Procedures 2
Plasmid construction 2
Plasmid compatibility 2
Phage expression 2
HER2 ELISA for functional analysis 2
Labeling reactions 3
In-gel fluorescence (IGF) 3
Western blot (WB) analysis and quantification 3
Supplementary Figures 4
ScFv Sequence 10
References 10
Author Contributions 10

Experimental Procedures

Plasmid construction

To construct the phagemids a gene block encoding 4D5 scFv with a spacer region was inserted via Gibson assembly between PelB and gIII in pADL-10b (Antibody Design Labs). The spacer region contains an HA tag peptide followed by a double arginine trypsin cleavage site (GSARRGS) and a Myc tag. The lacI operator was replaced via Gibson cloning by an orthogonal or a wild type ribosomal binding site. The first was amplified from RSF-D11_G9-GstCaM_1TAG and the second from RSF-wtRibo-wtRBS-GstCam.[1] Amber codons or quadruplet codons were inserted via site-directed mutagenesis.

pAux was created by replacing the origin of replication in pCDF PylRS/tRNA$_{CUA}$[2] by an RSF origin via Gibson cloning. PylRS, PylT and spectinomycin resistance were amplified from pCDF PylRS/tRNA$_{CUA}$[2] and the RSF origin of replication was amplified from in ribo-Q1 O-gst-cam.[1] A G-to-T point mutation was introduced into the RSF origin (RSF*) to increase the copy number of the plasmid as previously reported.[3] pAux/PylCUA was generated via Gibson assembly RiboQ1 and pAux. pAux was linearized and RiboQ1 was amplified from RSF-D11_G9_GSTCam.[1] pAux/Pyl$_{UACU}$ was prepared by linearizing pAux/Pyl$_{CUA}$ using PstI and SpeI and exchanging the WT PylT by an optimized PylT.[2] pAux/Pyl$_{UACU}$/Tyr$_{CUA}$ was generated by linearizing pAux/Pyl$_{CUA}$ using NotI and BamHI and inserting MjPrpRS and MjT between RiboQ1 and the spectionomycin resistance cassette. MjPrpRS/MjT was amplified from pKW MjPrpY_MjT,[2] digested with NotI and BamHI to generate sticky ends and ligated to linearized pAux/Pyl$_{UACU}$.

Plasmid compatibility

A protocol adapted from Phillips et al.[3] was followed. pAuxRQ1 and a phagemid encoding scFv(TAG127) were co-transformed into SS320 F' cells. Cells were grown from OD = 0.001 to OD = 2.0 in the absence of spectinomycin and plated in serial dilutions on LB-agar containing spectionmycin (50 µg/mL). Colonies were counted and the viability percentage was plotted.

Phage expression

Electrocompetent SS320 F' cells bearing either pAux/PylCUA, pAux/PylUACU, or pAux/PylUACU/TyrCUA were prepared. The corresponding phagemid was transformed by electroporation. Cells were plated on LB-agar supplemented with ampicillin 50 µg/mL (Amp), tetracycline and 5 µg/mL (Tet). Spectinomycin 37.5 µg/mL (Spec) was added to cells containing an auxiliary plasmid.
After overnight incubation at 37°C and 250 rpm, 5-10 colonies were inoculated in the same shaking flask overnight in medium containing Amp, Tet and Spec for cells containing the auxiliary plasmid and Amp and Tet for the rest. All media was supplemented with 2% glucose in order to inhibit the lacI promoter regulating the expression of the orthogonal ribosome.

Phage expression was performed as recommended by the provider of the helper phages (Antibody Design Labs) with some modifications. Briefly, after overnight incubation at 37°C and 250 rpm, the sample was diluted to OD = 0.25 and grown to OD = 0.5 in medium with the aforementioned antibotics without glucose. The OD was adjusted to 0.5 for all samples before adding the helper phage and 5 mL of each sample was superinfected with 5 µL of either CM13 or d3M13 from a commercial stock. A multiplicity of infection (MOI) of 14 was used as recommended by the provider unless otherwise indicated. Infection was allowed to proceed for 1 h at 37°C and 250 rpm. Subsequently, cells were centrifuged and the supernatant was discarded. Then cells were resupsended in medium containing kanamycin 50 µg/mL (Kan), Amp, and IPTG 1 mM. Spec was added to cells containing an auxiliary plasmid. CypK and PrpF were used at a final concentration of 2 mM except for dual incorporation, where both amino acids were used at 5 mM. Expression was performed at 30°C and 250 rpm for 18 h. After 18 h, phage was purified by two PEG/NaCl precipitation steps as previously reported[4] and redissolved in 50 µL of PBS.

Phage were counted by serial dilutions on ampicillin plates using a protocol adapted from Liu et al.[4] Briefly, for each sample 198 µL of SS320 F' cells at OD600 = 0.5 was added to a 96-well plate (triplicates) and infected with 2 µL of phage concentrated 100-fold from the expression medium. Cells were placed in a shaking incubator at 37°C for 1 h and subsequently diluted in PBS 10-fold 8 times in a 96-well plate. 5 µL of each well containing diluted sample was spotted on a 9-cm LB-agar plate with 100 µg/mL ampicillin and allowed to grow at 30°C for 16 h. The concentration of phage in the supernatant was calculated from the number of colonies present on spots containing 3-30 colonies.

HER2 ELISA for functional analysis

Functional binding of the anti-HER2 scFv was assessed by indirect ELISA. ELISA plates (Nunc 442404) were coated with 0.25 µg/mL of HER2 ectodomain (10001-HCCH. Sino Biologicals) in PBS overnight at 4°C or for 2 h at room temperature. Plates were washed

with PBS with 0.05% Tween 20 (PBST) 5 times after each step. Plates were blocked with 1% BSA in PBS for 1 h, washed with PBST, and samples were diluted 400 times and incubated 1-2h at RT. Subsequent to PBST washing, an anti-M13-pVIII–HRP conjugate antibody (GE Healthcare Lifescience 27-9421-01) was used for detection. Slow TMB (421101 BioLegend) was used as a substrate and the reaction was quenched with 1 M sulfuric acid. Absorbance was measured in a PHERAstar microplate reader at 450 nm. After subtracting background absorbance at 570 nm, the data were interpolated in a standard curve fitted using a 5-parameter logistic regression.

Labeling reactions

All labeling reactions were performed on phage in PBS concentrated 50x from the expression supernatant (0.1-10 pM).

Biotin-tetrazine: 0.8 µL of biotin-PEG4-tetrazine (CP-6001 ConjuProbe) from a 0.5 mM DMSO stock was added (10 µM final) to 20 µL of phage. The mixture was allowed to react for 12-16 h at 20-22ºC. After this time, reactions were quenched with 0.5 µL of 4 mM BCN-OH (100 µM final).

Tetrazine fluorophores: 1 µL of SiR-tetrazine (SC008 Biochrmoe) or Cy5-tetrazine (CLK-015 Jena Bioscience) from a 400 µM DMSO stock was added (20 µM final) to 20 µL of phage. The mixture was allowed to react for 12-16 h at 20-22ºC. After this time, reactions were quenched with 0.5 µL of 4 mM BCN-OH (100 µM final).

Azide fluorophores: 25x “click mixture” was prepared by mixing 1.5 µL of water, 1 µL of CuSO4 25 µM in water, 5 µL of tris(3-hydroxypropyltriazolylmethyl)amine (THPTA) ligand 25 µM in water, and 2.5 µL of sodium ascorbate 25 µM freshly prepared.[2] 1 µL of Cy7-azide (CLJ-1052 Jena Bioscience) from a 2 mM stock was added (100 µM final) to 20 µL of phage followed by 0.8 µL of “click mixture”. The components were allowed to react for 12-16 h at 20-22ºC.

In-gel fluorescence (IGF)

Samples were run on 4-12% Bis-Tris Nupage gels (Thermo Fisher Scientific) and imaged in an Amershan Typhoon Bimolecular Imager. Gels containing samples conjugated with Cy5-tetrazine, SiR-tetrazine or AF647-azide were imaged using an LD635 laser and Cy5 filter settings (λ_{ex} = 635 nm, λ_{em} = 670±15 nm). Gels containing samples conjugated with Cy7-azide were imaged using an LD785 laser and IRlong filter settings (λ_{ex} = 785 nm, λ_{em} = 825±15 nm).

Western blot (WB) analysis and quantification

Samples were denatured for 20 min in NuPAGE LDS protein loading buffer (Thermo Fisher Scientific) supplemented with 10% beta-mercaptoethanol. Samples were run on Nunc 4-12% bis-tris gels and tranfered to PVDF membranes using an iBlot 2 dry blotting system (Thermo Fisher Scientific). Membranes were blocked for 1 h in Odyssey blocking buffer (927-4000 LI-COR). All membranes were probed with anti-p3 (PSKAN3 MoBiTech) 1:2000 for 2 h at room temperature or overnight at 4°C. If samples were not biotinylated the antibody was diluted in PBS/blocking buffer 1:1 with 0.05% Tween 20. If samples were biotinylated, the anti-p3 antibody (1:2000) was co-incubated 800CW streptavidin (926-32230 LI-COR) 1:1000 overnight at 4°C in PBS/blocking buffer with 0.2% Tween 20 and 0.01% SDS. In all cases, after washing with PBST (PBS with 0.1% Tween20) 3 times 5 min, the membrane was probed with goat anti-mouse secondary antibody (either 680RD or 800CW LI-COR) 1:15000 for 1 h at room temperature. Samples were washed with PBST 3x 5 min and rinsed with water. Western blots were imaged with an Amersham Typhoon Biomolecular Imager or a LI-COR Odyssey Blot Imager. Bands were quantified through densitometric analysis using Image Studio and the signal was interpolated in a calibration curve using a linear fitting.

Supplementary Figures

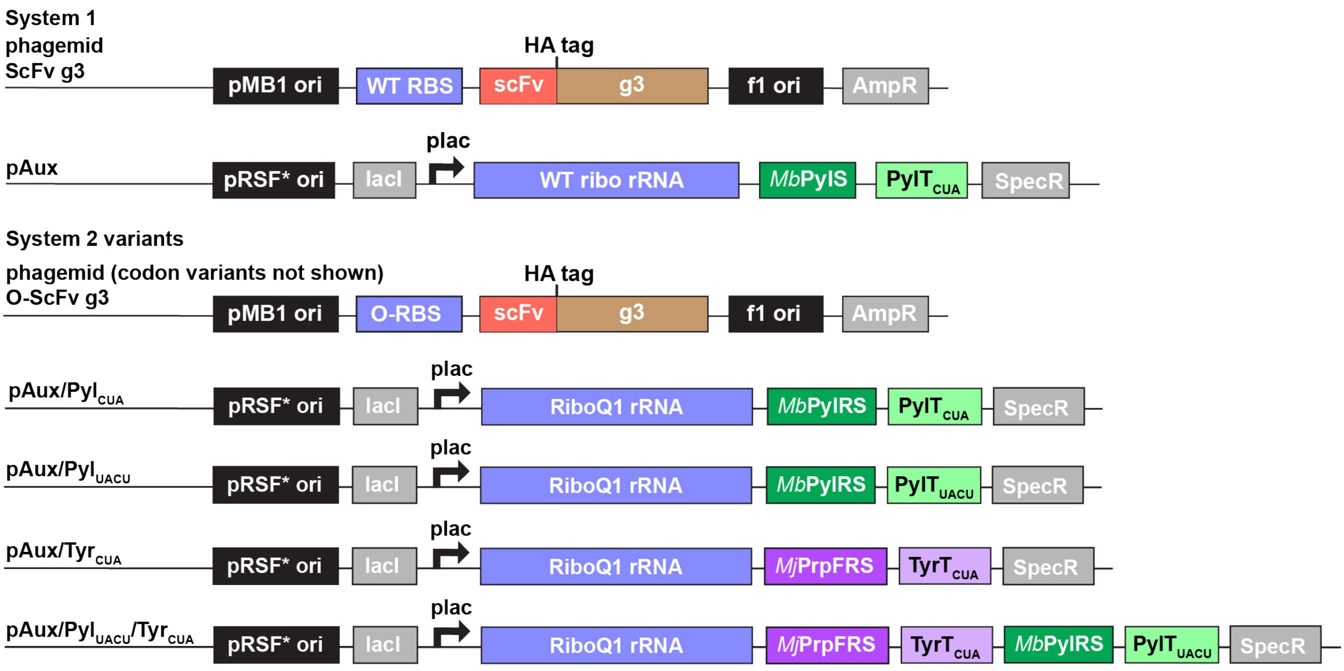

Figure S1. Selected regions of the plasmid systems used in this study.

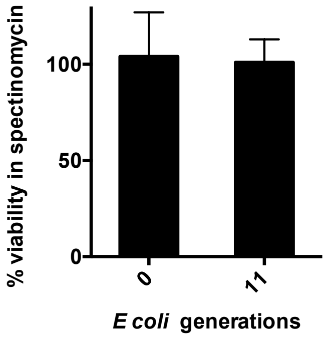

Figure S2. Stability of the auxiliary plasmid bearing the RSF mutant origin in the presence of the phagemid and the fertility plasmid. Viability of SS320 F' cells transformed with the phagemid encoding scFv(TAG252)-p3 was assessed on spectinomycin plates. Error bars represent the standard deviation of three biological replicates.

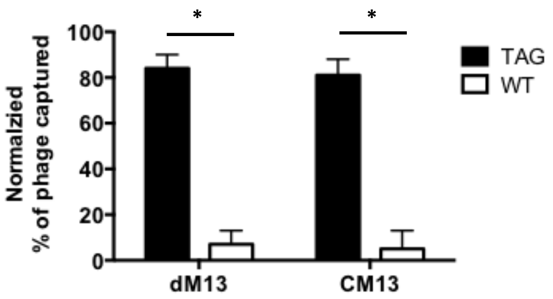

Figure S3. Over 80% of phage displaying ScFv-p3 bear reactive CypK when expressed with System 2 and a phagemid encoding O-ScFv(252TAG). This experiment was performed essentially as reported by Ng *et al.*[5]. A solution of phage was incubated with biotin-tetrazine to label reactive CypK. Then, labelled phage were depleted with streptavidin beads. The titer from phage remaining in solution after depletion (CFU2) and the titer before depletion (CFU1) were determined. The percentage of phage displaying reactive CypK was calculated as: [1-(CFU2/CFU1)]x100. The y axis in this plot represents the percentage of captured phage normalized by the average amount of phage displaying at least one ScFv-p3. This calculation provides the percentage of phages with ScFv-p3 that bear reactive CypK. Since the ratio of ScFv-p3/p3 in phage when using dM13 is 0.27 (see Figure S7), on average there is more than one ScFv-p3 per phage, thus the percentage of captured phage is divided by 1 for dM13. Since the ratio of ScFv-p3/p3 in phage when using CM13 is 0.12, there is 0.6 ScFv-p3 per phage on average, thus the percentage of captured phage is divided by 0.6 for CM13. Error bars represent the standard deviation from three biological replicates. These data indicate that 84 ± 6 % of phage displaying ScFv-p3 bears CypK when superinfected with dM13, which is similar to the 81 ± 6 % obtained when superinfected with CM13. (*$p < 0.01$)

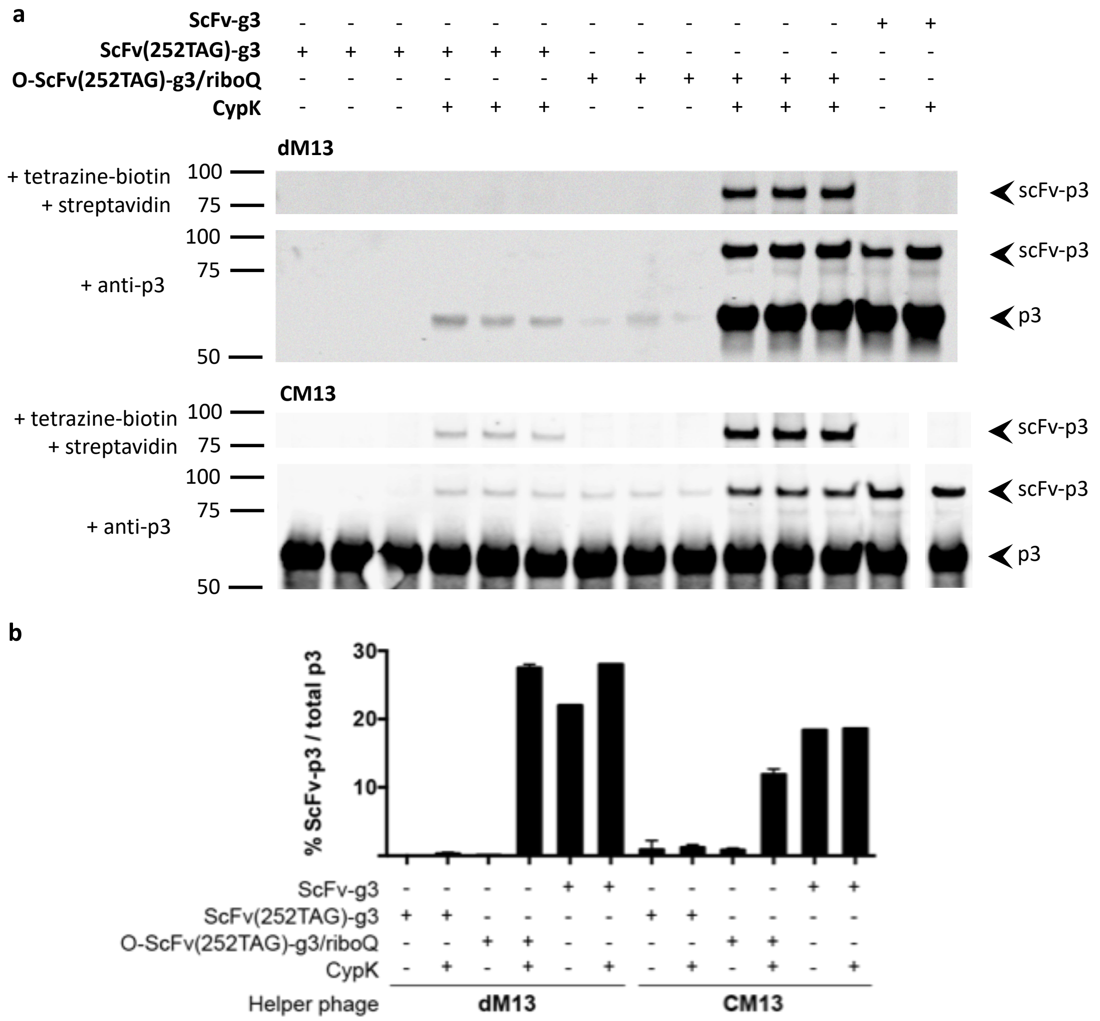

Figure S4. ScFv(CypK)-p3 and p3 production with dM13 and CM13. a) Western blot fluorescence shows ScFv-p3 labelled with tetrazine-biotin and probed with streptavidin, and ScFv-p3 and p3 probed with an anti-p3 antibody. See Figure 1e in the main text for quantification of the biotinylated ScFv-p3(CypK). b) Quantification of ScFv-p3/total p3. Error bars represent the standard deviation from three biological replicates. Percentage of ScFv-p3 vs total p3 for phages produced from O-ScFv(252TAG)-g3 is 27.5 ± 0.6 % with dM13 and 11.9 ± 0.9 % with CM13. Thus the levels of display in our systems are comparable to those in the parent phage display system. The display level from O-ScFv(252TAG)-g3 with CypK was the same as that from ScFv when using dM13. The display level of O-ScFv(252TAG)-g3 with CypK was 65% of that from ScFv when using CM13. Phage usually contains 5 copies of p3 per particle. These numbers indicate that, on average, phage from O-ScFv(252TAG)-g3 with CypK, expressed using dM13, bear 1 to 2 ScFv-p3. Phage from O-ScFv(252TAG)-g3 with CypK, expressed using CM13 bear one ScFv-p3 per two phage. In phage produced with the gene III knock-out helper phage (dM13) many p3 proteins do not display ScFv most likely due to proteolysis. ncAAs incorporation leads to wild-type levels of display (dM13), or near wild-type levels, of display (CM13), and ncAA incorporation leads to wild-type level dM13 titers for system 2 (Figure 1). These observations suggest that our system will have an advantage over previously reported systems for directed evolution experiments with ncAAs, in which there was a measurable bias against ncAA incorporation in selections.[6] Our data suggest that the expression bias against ncAA incorporation is minimized or eliminated; our systems should therefore perform favourably in directed evolution experiments.

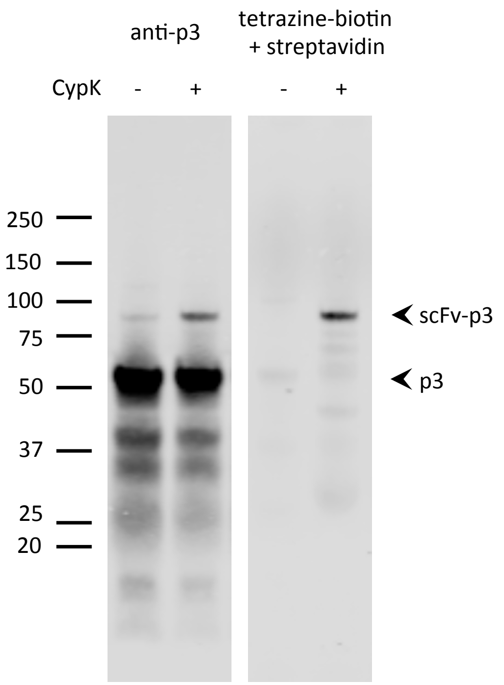

Figure S5. Selective labeling of scFv(G252)-p3 on bacteriophage expressed using CM13 and RiboQ1. Phage was conjugated with tetrazine-biotin and probed with fluorescent streptavidin and an anti-p3 antibody on a western blot.

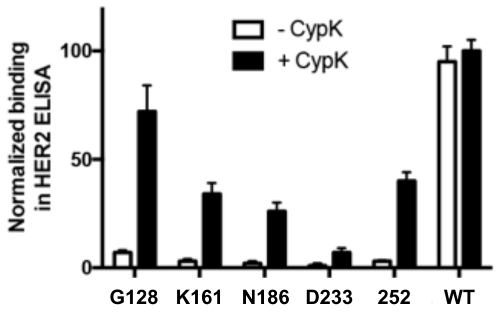

Figure S6. An scFv displayed on phage and with an amber codon at different positions binds its target HER2. Affinity was assessed by ELISA. Signal is expressed as a percentage of phage expressing WT scFv. Error bars represent the standard error from three biological replicates.

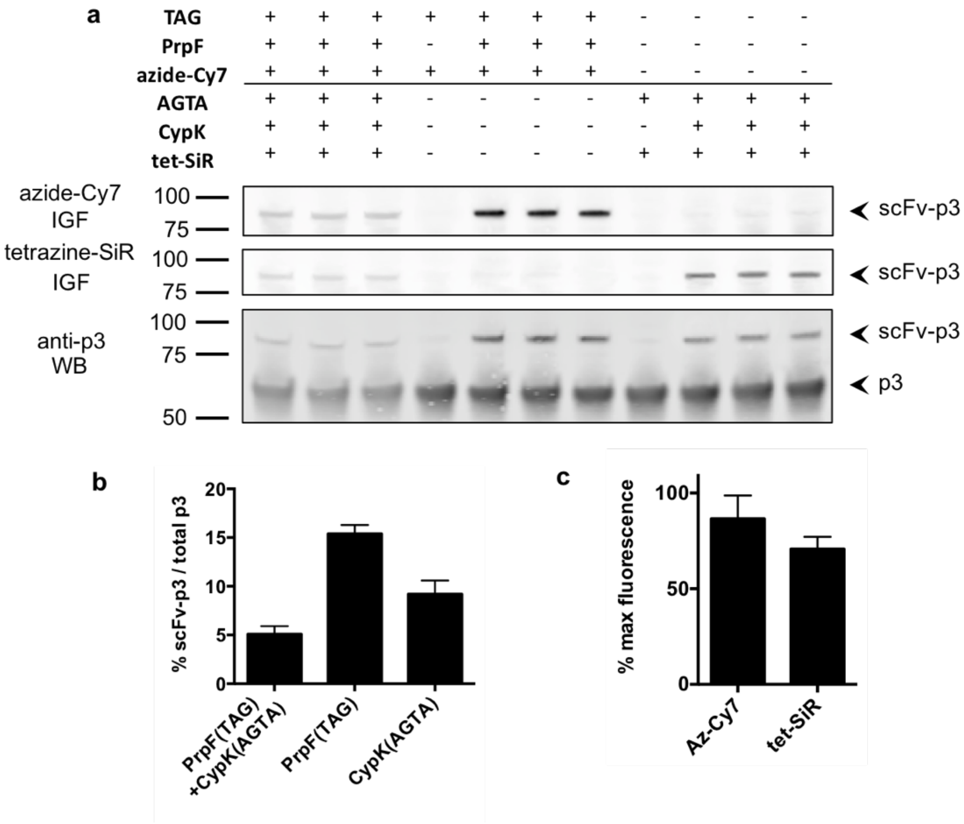

Figure S7. Quantification of dual ncAA incorporation into ScFv-p3. a) In-gel fluorescence of scFv-p3 dually modified with tetrazine-silicon-rhodamine (top, Cy5 channel) and picolylazide-sulfocyanine-7 (medium, Cy7 channel) after HA pull down. Western blot (bottom) probed with anti-p3 shows p3 and scFv-p3. IGF: in-gel fluorescence. WB: western blot. b) Quantification of the percentage of p3 displaying ScFv. The percentage of p3 displaying ScFv in the parental WT system is 22% (Figure S7). Thus the level of display in the single incorporation in response to a quadruplet codon is 46% that of WT; the level of display in the single incorporation in response to the amber codon is 84% that of WT and the level of display for proteins containing two ncAAs is 28% that of WT. c) The % of maximum fluorescence of the indicated fluorophore on ScFv-p3 from phage incorporating CypK and PrpF in response to amber and quadruplet codons. For Cy7, the "normalized maximum Cy7 fluorescence" is calculated by taking the Cy7 signal from labeling the phage incorporating PrpF in response to a single amber codon and dividing by the p3 signal in the corresponding western blot. The Cy7 signal for labeling phage incorporating CyPK and PrpF in response to amber and quadruplet codons is divided by the p3 signal in the corresponding western blot to calculate a "normalized Cy7 fluorescence for double incorporation". The % max fluorescence for Cy7 is calculated as: (normalized Cy7 fluorescence for double incorporation/ normalized maximum Cy7 fluorescence) x100. The % maximum fluorescence for SiR was calculated by an analogous procedure. All error bars represent the standard deviation from three biological replicates. The % max fluorescence values are Cy7: 86.6 +/- 12.2 %, SiR: 70.7 +/- 6.4 %. Thus, the majority of displayed ScFv is double labelled and contain both ncAAs.

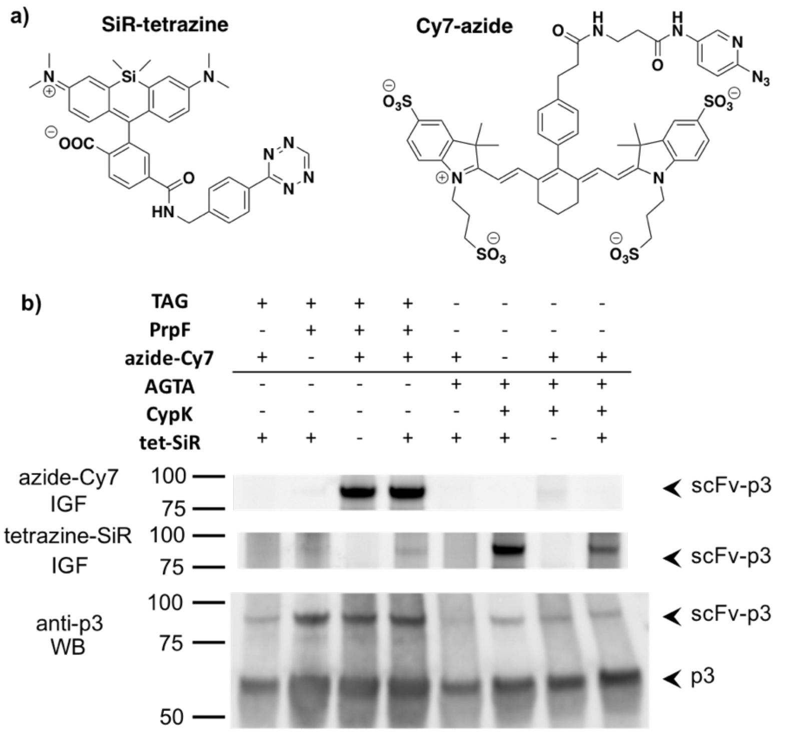

Figure S8. Selective conjugation of tetrazine-silicon-rhodamine (tetrazine-SiR) to CypK and pycolyl-azide-sulfocyanine-7 (azide-Cy7) to PrpF. a) Molecular structures of the two fluorophores. b) In-gel fluorescence is shown on the two top stripes, the far-red channel is shown above and the near-infrared below. scFv(PrpF127)-phage was incubated with either tetrazine-SiR, azide-Cy7 or both; labelling only took place in the presence of azide-Cy7, showing the selectivity of the labelling, and very low bleed-through on the far-red channel is observed. scFv(CypK252)-phage was incubated with either dye or both and was only labelled in the presence of tetrazine-SiR. Although labelling of scFv(CypK252) was diminished in the presence of both dyes, the band corresponding to scFv-p3 can be clearly identified. Western blot (bottom) probed with anti-p3 shows p3 and scFv-p3 is shown below as a loading control. IGF: in-gel fluorescence. WB: western blot.

ScFv-g3	-	-	-	-	+	+	-	-	-	-	+	+
ScFv(252TAG)-g3	+	+	-	-	-	-	+	+	-	-	-	-
O-ScFv(252TAG)-g3/riboQ	-	-	+	+	-	-	-	-	+	+	-	-
CypK	-	+	-	+	-	+	-	+	-	+	-	+
dM13	+	+	+	+	+	+	-	-	-	-	-	-
CM13	-	-	-	-	-	-	+	+	+	+	+	+
**Phage titer**												
replicate 1	$6.4\cdot10^{5}$	$4.8\cdot10^{7}$	$2.0\cdot10^{7}$	$1.1\cdot10^{9}$	$1.2\cdot10^{9}$	$1.4\cdot10^{9}$	$2.4\cdot10^{10}$	$2.0\cdot10^{10}$	$1.6\cdot10^{10}$	$8.0\cdot10^{9}$	$1.6\cdot10^{10}$	$8.0\cdot10^{9}$
replicate 2	$2.0\cdot10^{5}$	$3.6\cdot10^{7}$	$2.8\cdot10^{7}$	$1.1\cdot10^{9}$	$1.3\cdot10^{9}$	$1.2\cdot10^{9}$	$8.0\cdot10^{9}$	$1.2\cdot10^{10}$	$2.4\cdot10^{10}$	$2.4\cdot10^{10}$	$3.2\cdot10^{10}$	$2.4\cdot10^{10}$
replicate 3	$4.4\cdot10^{5}$	$3.2\cdot10^{7}$	$2.4\cdot10^{7}$	$1.0\cdot10^{9}$	$1.2\cdot10^{9}$	$1.4\cdot10^{9}$	$1.2\cdot10^{10}$	$2.4\cdot10^{10}$	$2.0\cdot10^{10}$	$8.0\cdot10^{10}$	$2.8\cdot10^{10}$	$2.0\cdot10^{10}$
**average**	$\mathbf{4.3\cdot10^{5}}$	$\mathbf{3.9\cdot10^{7}}$	$\mathbf{2.4\cdot10^{7}}$	$\mathbf{1.1\cdot10^{9}}$	$\mathbf{1.2\cdot10^{9}}$	$\mathbf{1.3\cdot10^{9}}$	$\mathbf{1.5\cdot10^{10}}$	$\mathbf{1.9\cdot10^{10}}$	$\mathbf{2.0\cdot10^{10}}$	$\mathbf{1.3\cdot10^{10}}$	$\mathbf{2.5\cdot10^{10}}$	$\mathbf{1.7\cdot10^{10}}$
SEM	$1.3\cdot10^{5}$	$4.8\cdot10^{6}$	$2.3\cdot10^{7}$	$2.3\cdot10^{7}$	$4.0\cdot10^{7}$	$9.3\cdot10^{7}$	$4.8\cdot10^{9}$	$3.5\cdot10^{9}$	$2.3\cdot10^{9}$	$5.3\cdot10^{9}$	$4.8\cdot10^{9}$	$4.8\cdot10^{9}$
**% HER2 ELISA signal**												
replicate 1	0.0	0.2	0.2	16.2	16.9	18.6	0.0	4.1	5.2	35.3	107.8	86.8
replicate 2	0.0	0.3	0.3	14.1	19.8	15.4	0.1	8.4	7.8	35.2	91.0	91.0
replicate 3	0.1	0.2	0.3	14.1	25.7	19.1	0.0	1.3	4.4	41.0	101.2	83.8
**average**	**0.0**	**0.2**	**0.2**	**14.8**	**20.8**	**17.7**	**0.0**	**4.6**	**5.8**	**37.2**	**100.0**	**87.2**
SEM	0.0	0.0	0.0	0.7	2.6	1.2	0.0	2.1	1.0	1.9	4.9	2.1
**% ScFv-p3(CypK) WB signal**												
replicate 1	0.0	0.5	0.0	25.9	-	-	1.9	10.0	3.2	104.2	-	-
replicate 2	0.0	1.1	0.1	23.6	-	-	1.7	15.9	2.8	92.8	-	-
replicate 3	0.1	0.6	0.1	29.4	0.4	0.5	1.4	14.5	1.6	103.0	1.2	1.5
**average**	**0.1**	**0.7**	**0.1**	**26.3**	**0.4**	**0.5**	**1.7**	**13.2**	**2.5**	**100.0**	**1.2**	**1.5**
SEM	0.0	0.2	0.0	1.7	-	-	0.1	1.8	0.5	3.6	-	-

Table S1. This table contains all numerical data for Figure 1. SEM: standard error of the mean.

ScFv Sequence

GACATCCAGATGACCCAGAGCCCTAGCAGCCTGAGCGCGAGCGTGGGCGATCGTGTTACTATCACCTGTCGTGCCTCTCAGGACGTTAACACCGCGGTAGC
ATGGTACCAGCAGAAACCGGGTAAGGCTCCAAAACTGCTGATTTATTCCGCGTCCTTTCTGTACTCTGGCGTTCCGAGCCGTTTCTCTGGTTCTCGTTCCGGC
ACTGATTTCACTCTGACCATCTCTTCTCTGCAACCGGAAGACTTCGCGACCTACTATTGCCAGCAACATTACACCACCCCACCTACTTTCGGCCAGGGTACCA
AAGTAGAAATCAAGCGTACGGTAGCTGGTGGCGGTGGTTCCGGTGGCGGTGGCAGCGGTGGCGGCGGTTCTGGTGGCGGCGGCTCCGAAGTTCAACTGGT
TGAGTCCGGCGGTGGTCTGGTGCAGCCGGGCGGTAGCCTGCGCCTGTCTTGCGCTGCGTCCGGCTTCAACATTAAAGACACCTACATTCACTGGGTCCGTC
AGGCTCCGGGCAAGGGTCTGGAATGGGTAGCGCGCATCTACCCGACCAACGGTTATACCCGCTACGCAGATTCTGTTAAAGGTCGCTTTACCATTTCCGCAG
ACACCAGCAAAAACACCGCTTACCTGCAAATGAATTCTCTGCGCGCAGAAGATACTGCTGTATATTACTGTAGCCGTTGGGGCGGTGATGGTTTCTACGCAAT
GGATTACTGGGGTCAGGGTACTCTGGTTACTGTGAGCTCTXXX*GGATCCGAACAAAAACTCATCTCAGAAGAGGATCTGGGTAGCGCACGTCGGGCAGGTTC
ATATCCGTATGATGTTCCGGATTATGCAAGCGGT*

Codons replaced by amber codons or quadruplets are underlined. Position XXX is absent in the wild type ScFv.

Spacer region downstream of the ScFv and immediately before g3 is represented in italics.

References

[1] K. Wang, A. Sachdeva, D. J. Cox, N. M. Wilf, K. Lang, S. Wallace, R. A. Mehl, J. W. Chin, *Nat. Chem.* **2014**, *6*, 393.
[2] A. Sachdeva, K. Wang, T. Elliott, J. W. Chin, *J. Am. Chem. Soc.* **2014**, *136*, 7785-7788.
[3] G. J. Phillips, S. K. Park, D. Huber, *BioTechniques* **2000**, *28*, 400-408.
[4] C. C. Liu, A. V. Mack, M.-L. Tsao, J. H. Mills, H. S. Lee, H. Choe, M. Farzan, P. G. Schultz, V. V. Smider, *Proc. Natl. Acad. Sci. USA* **2008**, *105*, 17688-17693.
[5] S. Ng, M. R. Jafari, W. L. Matochko, R. Derda *ACS. Chem. Biol.* **2012**, *7*, 1482-1487.
[6] C. C. Liu, A. V. Mack, M.-L. Tsao, J. H. Mills, H. S. Lee, H. Choe, M. Farzan, P. G. Schultz, V. V. Smider, *Proc. Natl. Acad. Sci. USA* **2008**, *105*, 17688-17693.

Author Contributions

B.O.-S. Conceptualization: Equal; Data curation: Lead; Formal analysis: Lead; Investigation: Lead; Methodology: Lead; Project administration: Equal; Writing—original draft: Equal; Writing—review & editing: Equal.

J.C. Conceptualization: Equal; Funding acquisition: Lead; Project administration: Equal; Supervision: Lead; Writing—original draft: Equal. Writing—review & editing: Equal.

*Current address of B.O.-S. is: IQS School of Engineering – Ramon Llull University. Via Augusta 390, 08017 Barcelona. Spain.
